# The Potential Role of Vaccines in Preventing Antimicrobial Resistance (AMR): An Update and Future Perspectives

**DOI:** 10.3390/vaccines11020333

**Published:** 2023-02-01

**Authors:** Vincenzo Costanzo, Giovanni N. Roviello

**Affiliations:** 1 Department of Experimental, Diagnostic and Specialty Medicine, University of Bologna Alma Mater Studiorum, 40126 Bologna, Italy; 2 Italian National Council for Research (IBB-CNR), Area di Ricerca site and Headquartes, Via Pietro Castellino 111, 80131 Naples, Italy

**Keywords:** vaccines, antibiotics, pathogens, infectious diseases, antimicrobial resistance, prophylaxis, tuberculosis, enteric fever

## Abstract

In the modern era, the consumption of antibiotics represents a revolutionary weapon against several infectious diseases, contributing to the saving of millions of lives worldwide. However, the misuse of antibiotics for human and animal purposes has fueled the process of antimicrobial resistance (AMR), considered now a global emergency by the World Health Organization (WHO), which significantly increases the mortality risk and related medical costs linked to the management of bacterial diseases. The current research aiming at developing novel efficient antibiotics is very challenging, and just a few candidates have been identified so far due to the difficulties connected with AMR. Therefore, novel therapeutic or prophylactic strategies to fight AMR are urgently needed. In this scenario, vaccines constitute a promising approach that proves to be crucial in preventing pathogen spreading in primary infections and in minimizing the usage of antibiotics following secondary bacterial infections. Unfortunately, most of the vaccines developed against the main resistant pathogens are still under preclinical and clinical evaluation due to the complexity of pathogens and technical difficulties. In this review, we describe not only the main causes of AMR and the role of vaccines in reducing the burden of infectious diseases, but we also report on specific prophylactic advancements against some of the main pathogens, focusing on new strategies that aim at improving vaccine efficiency.

## 1. Introduction

The advent of antibiotics in the middle of the last century has provided clinicians with a powerful weapon to treat and prevent infectious diseases, allowing them to save millions of lives worldwide. The medical interventions that counteract the bacterial infections available before the antibiotic era included just weak and ineffective options, such as surgical drainage or spontaneous cures. As a consequence, death occurred very easily following pneumonia or endocarditis infections [1]. Since their discovery, antibiotics have been successfully employed by physicians to halt the progression of several infections. In this respect, different classes of antibiotics are available according to the strain of infecting pathogens and the stage of the disease [2]. Consequently, the morbidity and mortality caused by bacterial infections, as well as the healthcare-related costs, have been significantly reduced over the last few decades thanks to the availability of effective antibiotics. Despite the outstanding therapeutic benefits offered by these drugs, the enthusiasm of clinicians has progressively diminished over the last few years as a serious concern related to their effectiveness has emerged. This is, in fact, seriously affected by antimicrobial resistance (AMR), which is the ability developed by pathogens to resist the drugs intended to kill them. AMR is particularly alarming as it may cause persistent infections, as well as increased patient mortality and medical costs, and for this reason, is nowadays regarded as a serious global emergency. According to recent estimates, the average annual deaths due to AMR will exceed those due to cancer by 2050 [3]. This clearly explains the increasing attention paid to new cures and prophylactic interventions for contrasting microbial diseases and resistance phenomena. However, current efforts for the identification of new efficient antibiotics are not sufficient to overcome the threat represented by AMR. Even though the use of antibiotics is still crucial in the presence of a number of infectious diseases, other solutions that aim at eradicating resistant pathogens are imperative. In this context, vaccines hold a great potential to diminish the spread of highly pathogenic microbes and to minimize the necessity of antibiotics: the main cause of AMR. However, currently, there are no vaccines available for several resistant pathogens, and their applicability is hampered by numerous obstacles encountered by scientists and companies.

In this article, we first explore the main causes originating in AMR and discuss the difficulties related to antibiotic development. Thereafter, we describe the role of vaccines in fighting the rise of AMR as well as the current advancement of preclinical and clinical vaccine production against the main resistant pathogens. In addition, we report on the innovative prophylactic options available to augment vaccine efficiency in the fight against pathogens and AMR.

## 2. Main Causes of Antimicrobial Resistance

The abuse and misuse of antibiotics in humans are among the main causes that have led to AMR. The implementation of these drugs for the neutralization of viral respiratory infections represents a typical example of antibiotic misuse that is driving the spread of resistant pathogens and their determinants [4]. This is largely due to the possibility of having access to antibiotics without any medical prescriptions, which occurs in most developing countries such as Africa [5] and the Philippines [6]. Purchasing antimicrobials in pharmacies, in fact, allows one to avoid long waiting times and to discuss directly with the seller about therapeutic options, thus escaping counseling fees. However, the vendors are often untrained people and may not be aware of the drug’s contraindications and the dosage required for specific patients.

The storage conditions of antibiotics represent another factor contributing to AMR. Indeed, the quality of any drug in terms of efficacy can be significantly compromised if the storage temperature and humidity are extremely high. As reported, the antimicrobials commercialized in Africa may degrade very easily because of adverse weather conditions. Since degraded drugs contain less active principle, patients treated with antimicrobials often do not reach the optimal drug concentration, thus producing no effective results following their consumption.

This consequent under dosage inevitably causes therapeutic failure and fuels the resistance of several strains of bacteria [7,8]. A further determinant of increased AMR is the inappropriate prescription of antibiotics by doctors, which includes erroneous drug choice and incorrect dosage, eventually leading to the uselessness of pharmacological treatment. As shown in a pilot study performed on 270 patients from different regions of Lebanon, about half of the antibiotic prescriptions over a period of four months resulted in inappropriate use and did not conform to the Infectious Disease Society of America (IDSA) guidelines, hence reflecting the variability in medical knowledge, the lack of clinical trials aimed at optimizing treatment duration, as well as the social factors of the country involved in medical decisions [9].

It is well-reported that the correct choice of antibiotics for a given infection is crucial to eradicating the pathogens and, in turn, to rise patient survival [10]. Drug selection can be facilitated by performing an antibiotic susceptibility test (AST), an essential surveillance assay by which the susceptibility of any pathogens to specific antimicrobials is measured [11]. However, the AST is a time-consuming procedure that may require the implementation of many approaches, such as bioluminescence, chemiluminescence, and flow cytometry, raising the costs of analysis [12]. Obviously, most laboratories in rural places are not equipped to perform such analyses. In cases where AST is not possible, most health professionals are forced to prescribe broad-spectrum antibiotics instead of more specific ones in an attempt to treat infectious diseases, which significantly contributes to increasing the burden of AMR [13]. This is made worse by the medical prescription of multiple antibiotics for treating the same infection following both the lack of a specific diagnosis and the economic advantages coming from drug suppliers. Furthermore, the huge number of patients usually under care by family doctors limits the time required for their optimal training with respect to the knowledge of a given disease and the available therapeutic options. Even more alarmingly, patients often rely on self-medication, which in turn leads to therapy failure as a result of unjustified drug discontinuation or dosage changes. This is particularly true when patients experience symptom relief, typically occurring before a pathogen is completely eradicated [14]. Antimicrobials are also employed as prophylactic agents to prevent infectious diseases and as chemotherapy drugs in high-risk animals, as well as those that are exploited to facilitate the growth of livestock. Noteworthy, the amount of antimicrobials used for these purposes greatly exceeds that used for human therapy [15].

The continuous and massive administration of these drugs in animals stimulates the spread of resistant bacteria that can be transmitted to humans after food consumption, contact with animals, or through the environment [16]. As demonstrated by the high levels of antimicrobials residues found in meat for consumption and its derivatives, livestock represents a major source of resistant pathogens, which are more likely to be transmitted to humans, especially in countries where people live in close contact with animals [17,18]. It has been proven that the misuse of antibiotics in domestic animals for zootechnical use may lead to new mutations in the bacterial genome. This phenomenon, together with the transmission/acquisition of plasmids, allows pathogens to escape antimicrobial action which usually occurs through drug detoxification, target modification, or changes in metabolic pathways [15]. Alterations in target biomolecules can greatly affect molecular binding with antimicrobials. Such changes can involve targets such as the ribosomal 30S and 50S subunits but also cell wall precursors and enzymes with crucial roles in RNA transcription or DNA replication [19].

**Figure 1:**
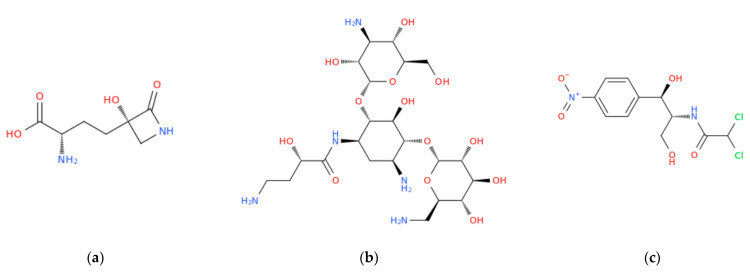
Structural representation of three examples of antibiotics taken from the three main families herein mentioned: tabtoxinine beta-lactam, a beta-lactam antibiotic derived from tabtoxin (**a**); amikacin, a broad-spectrum semi-synthetic aminoglycoside antibiotic, derived from kanamycin (**b**); chloramphenicol, a semisynthetic broad-spectrum antibiotic derived from *Streptomyces venequelae* (**c**).

**Figure 2:**
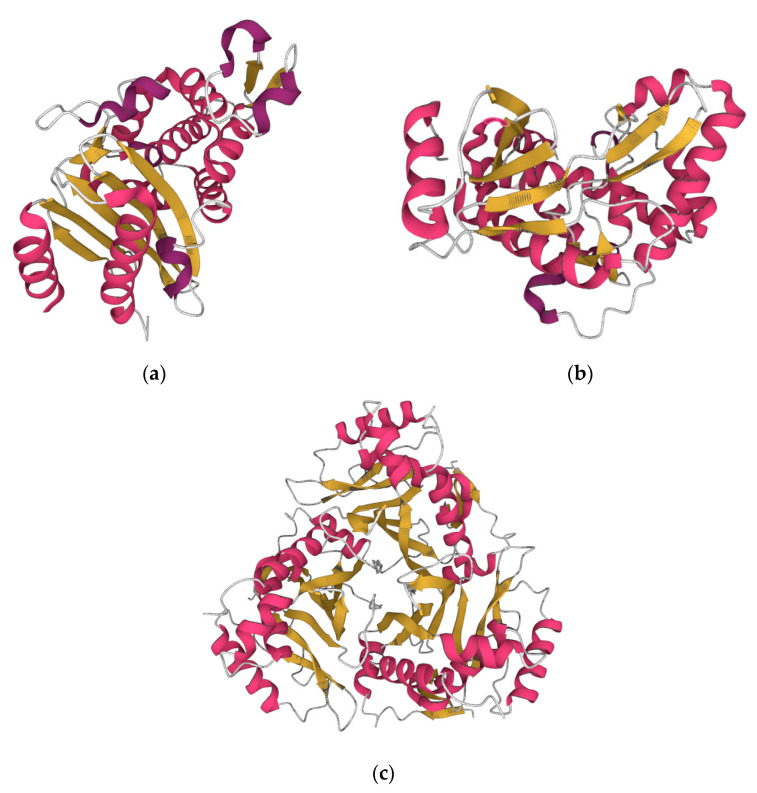
The beta lactamase of *Vibrio parahaemolyticus* (https://www.rcsb.org/structure/6IZC accessed on 13 December 2022) (**a**). The aminoglycoside-modifying enzyme APH(2″)—IVa (aminoglycoside phosphotransferase 2″—IVa) of *Enterococcus casseliflavus* (https://www.rcsb.org/3d-view/3N4V/1 accessed on 13 December 2022) (**b**). The type III chloramphenicol acetyltransferase of *Escherichia coli* (https://www.rcsb.org/3d-view/3CLA/1, accessed on 13 December 2022) (**c**).

Overall, pathogens have learned how to inactivate several classes of antibiotics. Among these, beta-lactams, aminoglycosides, and chloramphenicol (Figure 1) are inhibited through the use of three main key enzymes: beta-lactamases, aminoglycoside-modifying enzymes, and chloramphenicol acetyltransferases, respectively (Figure 2), which clearly leads to dramatic scenarios of AMR [19]. As above mentioned, the misuse of antibiotics has generated a plethora of resistant pathogens that easily escape the antimicrobial attack. Therefore, research is constantly focused on the identification of novel compounds that may be effective in treating such microbes.

## 3. Challenges in Developing New Antibiotics

Despite the great efforts made by researchers and companies to develop new antimicrobial drugs, only a few molecules have been recognized so far as effective antibiotic candidates. In fact, the number of new antimicrobials developed later than the 90s has progressively diminished, and many of them correspond to slight modifications of existing drugs [20,21]. The search for new antimicrobials is challenging, and this can be due to several factors, which are mainly classifiable as scientific and commercial difficulties [22].

The first challenge that companies and investigators have to face in the development of lead molecules is the need for compounds that are effective in inactivating or killing bacteria through specific processes. The results obtained by GlaxoSmithKline (GSK), which evaluated more than 300 bacterial genes and performed about 70 high throughput screenings from 1995 to 2001, were very disappointing, with a success rate lower than 10% [23]. Similar results were reported by several other profitable companies, including AstraZeneca, and huge research costs have been sustained without any satisfactory outcomes.

Moreover, optimizing the antimicrobial effect of lead molecules constitutes a task that is not easy to achieve. In the case of Gram-negative pathogens, for example, an effective antimicrobial is expected to penetrate the outer membrane and the cell wall of the bacteria to reach the intracellular target and exert the desired effect. These layers are endowed with different properties and lipophilicity, which makes the design and realization of a desirable compound particularly difficult. Not less importantly, the designed drug should be able to avoid the bacterial efflux pump, a mechanism by which the drug could be pumped out from the bacteria without reaching the target [22].

Another difficulty is represented by the optimization of the safe and tolerable properties of a drug. This is usually a very prolonged process that is affected by different factors. In the case of antimicrobials, adverse events are a major safety concern, with antibiotics being implicated in ∼20% of all drug-related emergency department visits, as reported in studies conducted in the United States [24]. Most of these visits are due to allergic reactions accounting for 79% of all the adverse effects of antibiotics; however, other conditions, including gastrointestinal, psychiatric, and/or neurologic symptoms are also associated with antimicrobials [24]. The chemical nature of the compound, is reconsidered and modified when a safety issue is observed during a lead identification program, causing further time and cost consumption. In addition to these concerns, the conduction of clinical trials to test antimicrobials in vivo is generally hampered by several factors. For example, the recruitment of new patients involved in clinical trials requires that they have not been treated with antimicrobials 24 h before starting the trial. However, this often represents an insurmountable barrier as some patients, especially those with advanced pneumonia, need to assume antibiotics as soon as possible in order to reach high survival rates. As a consequence, the clinical study is greatly delayed [22].

Among the major challenges stalling new antimicrobial development, it is also worth mentioning the lack of funding needed to support expensive clinical trials, but even more importantly, of a market interested in newly licensed antibiotic drugs [25]. To overcome these difficulties, there is an urgent need for commitment to national funding and international coordination on this theme. Sadly, governments of many countries are reluctant to invest significant resources in the fight against antimicrobial resistance that they see as an issue that is not solely their own. On the other hand, low-resource countries, where AMR has the highest burdens, have fragile economic conditions that do not let them lead the reform of the antimicrobials discovery and market [25].

## 4. Effect of Vaccines on AMR

Hence, other strategies aimed at fighting AMR have been proposed [26]. Among these, vaccines may be the key to limiting the spread of resistant pathogens and the consequent AMR. Vaccines have represented an important achievement in the field of immunology since their implementation in the prophylaxis therapy of patients worldwide contributes significantly to the prolongation of life expectancy. The importance of vaccination has recently emerged with the COVID-19 pandemic, for which the fast development of safe and efficient vaccination was an urgent necessity [27,28].

After vaccination, the resulting production of antibodies is specific to eradicate a certain pathogen for a time whose length depends on the type of vaccine, and this immune response is different among individuals. Vaccines and antibiotics have different mechanisms of action, and this results in a much lower probability of developing resistance after vaccination. Since they are prophylactic agents, vaccines work efficiently before pathogens replicate and spread in different organs. This is crucial to minimize the likelihood of drug resistance caused by mutations in the pathogen genome [29]. As a proof of concept, the effect of vaccination against Streptococcus in the USA reduced by 84% the cases of *Streptococcus pneumonia* caused by multidrug resistance in children younger than 2 years of age (Table 1) [30]. While an antibiotic is designed against a specific target, vaccines are directed against multiple targets, which makes resistance episodes in the vaccinated population very rare. In fact, more mutations conferring resistance are needed if multiple immunogenic epitopes are exposed [26]. Additionally, the duration of the protection and the herd immunity, if reached, make the vaccines more efficient and reliable tools than antibiotics. According to the above-mentioned study [30], vaccination reduced the transmission of *Streptococcus pneumonia* in unvaccinated 65-year-old people by 49% (Table 1). Even in the rare case of a resistance to vaccines, such as the three examples reported by Kennedy and Read [31], two of which involved vaccination against bacterial pathogens (*Streptococcus pneumonia* and *Bordetella pertussis*), a severe outcome of the disease is still avoided due to the preventive nature of vaccines. On the contrary, the antimicrobial therapeutic effect is completely blocked in the case of resistance to antibiotic drugs [29,31].

Famously, vaccines permitted the eradication of smallpox [33] and rinderpest virus [34], almost eliminated poliomyelitis, and significantly contributed to reducing the incidence of other diseases, including pertussis, tetanus, and diphtheria [35].

The reason why vaccination can be a successful strategy for the treatment of AMR lies in the evidence that vaccines can directly restrain the spread of resistant pathogens through specific antibodies, thus minimizing or avoiding the usage of antibiotics. Such effects have been well documented with *Haemophilus influenzae* type b, Pneumococci, meningococci, and Rotavirus vaccines [36]. According to a clinical trial carried out in 2018 on 6–35 months old children, individuals receiving quadrivalent Influenza vaccines showed a 47% lower incidence of influenza compared to the placebo group (Table 1). This outcome was accompanied by a 50% reduced antibiotic prescription [32].

Similar results were obtained in Ontario, where the vaccination of children against influenza led to a 64% reduced antibiotic prescription with respect to other provinces of Canada [37]. An indirect effect of vaccines is to prevent secondary bacterial infections, such as pneumonia and otitis, which can easily occur following viral infection. By inhibiting secondary infections after vaccination, the inappropriate consumption of antibiotics is averted. As described in a single-blind study by Ozgur et al., 119 Turkish children who underwent influenza vaccination experienced a significant reduction in acute otitis media, otitis media with effusion, and total otitis compared to the unvaccinated group, confirming the usefulness of vaccination in preventing secondary infections [38].

## 5. Current Vaccines in Preclinical and Clinical Development

In their work, Micoli et al. listed the antimicrobial-resistant pathogens described as critical by the World health organization (WHO) and Centers for Disease Control and Prevention (CDC), together with the diseases they cause and the antibiotics to which they have developed resistance [26]. On 12 July 2022, the first report on the pipeline of vaccines in the course of development to mitigate the AMR has been published by WHO, stating that the clinical trials of last-stage development vaccines should be speeded up, and the implementation of existing vaccines boosted (https://www.who.int/news/item/12-07-2022-urgent-call-for-better-use-of-existing-vaccines-and-development-of-new-vaccines-to-tackle-amr accessed on 9 December 2022). This report describes not only vaccines in preclinical and clinical development but also failed candidates. Moreover, it classifies pathogens into different groups according to the feasibility expressed as the progression of the candidate vaccine in clinical trials, its biological as well as product development feasibility, and access and implementation feasibility. The biological feasibility takes into account factors including immunity from natural exposure and the likelihood of a vaccine to protect against most pathogenic strains. On the other hand, the product development feasibility relies on the availability of in vivo and in vitro models to assist vaccine development, the set-up of a late-stage clinical test, and the usage of human models, if required. Access and implementation feasibility refer to the possibility of easily introducing novel vaccine candidates in a target population, such as through children’s vaccination programs, by means of commercial incentives and political transparency. *Pseudomonas aeruginosa*, *Klebsiella pneumoniae*, *Extraintestinal pathogenic Escherichia coli* (ExPEC), *Acinetobacter baumannii,* and *Enterotoxigenic Escherichia coli* (ETEC), which were listed as critical priority pathogens, have shown 4, 5, 4, 5, and 10 vaccine candidates, respectively. For *Staphylococcus aureus*, a high-priority pathogen, there were 14 candidates. The highest number of candidates (20), however, has been found in the case of *Mycobacterium tuberculosis*, which is a well-established priority pathogen. The numbers mentioned above refer to preclinical analyses carried out in 2021, where a total of 94 candidates were confirmed. (Table 2), (https://www.who.int/news/item/12-07-2022-urgent-call-for-better-use-of-existing-vaccines-and-development-of-new-vaccines-to-tackle-amr accessed on 9 December 2022). A total of 61 candidate vaccines were confirmed in active clinical trials. *S. pneumonia* and *M. tuberculosis* have the highest number of candidates (16 and 13, respectively). *K. pneumoniae* and *Neisseria gonorrhoeae* both have one candidate vaccine. Instead, no vaccine candidates have been identified for *P. aeruginosa*, *Helicobacter pylori*, *Campylobacter jejuni*, *Enterobacter* spp., *Enterococcus faecium,* and *A. baumannii* (Table 2). Remarkably, the high and critical-priority pathogens tend to show fewer vaccine candidates compared to the medium-priority ones both in preclinical and clinical trials. Vaccines against *Shigella sonnei* and *H. pylori* have been discontinued, reflecting a common issue of vaccine production represented by economic, logistic, and scientific obstacles.

As above-mentioned, a classification of pathogens according to the feasibility of vaccine candidates and their development state has been created. Group A includes pathogens for which a vaccine has been licensed, while pathogens with vaccine candidates in clinical trials belong to group B. Group C and D include, respectively, feasible pathogens but with challenges in targeting the vaccine and pathogens with low feasibility in vaccine production (https://www.who.int/news/item/12-07-2022-urgent-call-for-better-use-of-existing-vaccines-and-development-of-new-vaccines-to-tackle-amr accessed on 9 December 2022).

### 5.1. Group A

#### 5.1.1. *Salmonella enterica ser. Typhi*

In recent years more than 20 vaccines have been brought to market against *S. enterica ser. Typhi*. They are related to three main vaccines: oral live attenuated Ty21a, unconjugated Vi polysaccharide (ViPS), and the typhoid conjugate vaccine (TCV). However, many drawbacks have been found with these vaccines, including a lack of immunogenic memory, a short-term duration of the antibody response, and limited effectiveness in young children [39]. For this reason, the scientific community has devoted its efforts to new strategies aimed at overcoming the burden of Salmonella disease by developing novel candidates.

Among the vaccines currently in development, eight are in preclinical trials, whereas five are in clinical trials. Modified Ty21a, combined invasive non-typhoidal Salmonella (iNTS)- generalized-modules-for-membrane-antigens (GMMA), and iNTS-TCV which are preclinical trivalent vaccines targeting different serotypes, such as *Salmonella enterica ser. Typhi*, *Shigella flexneri*, *Shigella sonnei*; non-typhoidal *Salmonella (NTS) enterica ser. Typhimurium,* and *Enteritidis*, in addition to *S. enterica ser. Typhi*. Another three bivalent candidates targeted *S. enterica ser. Typhi* and *S. enterica ser. Paratyphi A*. A novel Vi polysaccharide conjugated with diphtheria toxoid (Vi-DT conjugate vaccine) proved to be safe and immunogenic during a phase I study performed in Indonesia (NCT03109600), and it was able to produce high geometric mean titers (GMT) in adults and young children just after a single dose of the vaccine [40]. Phase II trials of this candidate (NCT03527355) showed high safety and immunogenicity in 6–23 years old children after 6 months from the vaccination with the first and second doses [41]. Currently, it is involved in phase III with the purpose of obtaining qualification from WHO and becoming a crucial vaccine for global health. A glycoconjugate trivalent candidate triggering *NTS enterica ser. Typhimurium,* and *Enteritidis*, and typhoid are actually under evaluation in a phase I trial in the United States (NCT03981952) [42]. Another candidate is represented by M01ZH09, a live attenuated oral vaccine directed against *S. enterica ser. Paratyphi A* and *Typhi*. The phase II trial proved that M01ZH09 was effective in reducing the risk of typhoid infection by 50% in patients where anti-Vi antibodies were found before the vaccination. (NCT01405521). Additionally, M01ZH09 was characterized by high immunogenicity and high tolerability and was able to greatly delay the onset of infection and reduce noticeably the burden of infection [43]. Typhax was an additional candidate vaccine that exploited a matrix to entrap Vi polysaccharide from *S. enterica ser. Typhi*. In a previous phase I study (NCT03926455), Typhax was demonstrated to be safe, immunogenic, and well-tolerated following a single dose. Nevertheless, a further boost in antibody levels was not reached with a second dose [44]. A very recent trial carried out on mice, rabbits, and monkeys showed that Advax-CpG adjuvant boosts the Vi antibodies response to Typhax compared to the previous formulation, hence, giving durable and sustained protection over time [45].

#### 5.1.2. *Mycobacterium Tuberculosis*

Bacillus Calmette-Guérin (BCG), developed in 1921 and included in 1974 in the WHO Expanded Program on Immunization (EPI), is, so far, the most used licensed vaccine against *M. tuberculosis*. The BCG vaccine was able to prevent tuberculous, meningitis, and tuberculosis cases in children born in 2002 when administered at birth or shortly after, showing a worldwide coverage of 76% [46]. Despite the good results demonstrated in preventing tuberculosis disease, this attenuated vaccine shows a wide variability, confirmed by the absence of immunity found in the Indian population compared to 80% of efficiency among the British population. The different strains, as well as the route of administration, have been shown to affect the levels of immunization and, in turn, the efficacy of vaccination [47]. Indeed, the percutaneous inoculation of BCG in Japanese people led to a higher production of BCG-specific CD4+ and CD8+ T cells compared to intradermal administration in Danish people [48]. In addition, the different strains of BCG caused an evident heterogeneity in the formation of crust, redness, and erythema in different countries, showing that genetic factors are crucial for the immunity system [47]. While the BCG vaccine is largely employed in those countries with a high-risk population, a selective vaccination is preferred in low to moderate-risk countries [49]. Therefore, new strategies in BCG vaccination are needed in order to obtain safe and efficacy vaccines in all groups. Currently, there are more than 20 candidate vaccines in preclinical studies and 13 novel candidates in active clinical trials. Among these, nine are prophylactic vaccines, while three have exclusive therapeutic effects. Another candidate has both effects. VPM1002 is a recombinant (r)BCG vaccine, genetically modified at the Max Plank institute, that reached phase 3 trials and showed better immunogenicity and safety compared to BCG (NCT04351685). This vaccine candidate is scheduled to complete phase 3 in 2025.

Another candidate under development is MTBVAC, a live attenuated vaccine containing all the antigens of *M. tuberculosis*. In a previous phase 1 studies, MTBVAC proved to be safe and immunogenic in both adults [50] and infants [51], demonstrating the possibility of a promising candidate. Additionally, the administration of MTBVAC to rhesus macaques (*Macaca mulatta*) in preclinical studies was well tolerated and gave similar results obtained in humans during clinical trials of the same candidate [52]. The good results achieved during phase 2 trials conducted in Africa on infants (NCT03536117), as well as on adolescents and adults (NCT02933281), allowed MTBVAC to enter the phase 3 evaluation where it is currently involved (NCT04975178) [53]. MIP/Immuvac is a heat-killed *Mycobacterium indicus pranii* vaccine that is currently involved in Phase 3 trials in India (CTRI/2019/01/017026). The first purpose of this study is the efficacy assessment of this candidate by evaluating the incidence of tuberculosis among healthy Indian people in contact with sputum-positive pulmonary tuberculosis patients over 3 years. Clinical reports demonstrated the high tolerability and efficacy of the MIP vaccine in preventing and treating pulmonary tuberculosis in a double-blind, randomized multi centric clinical study [54]. An immunogenic fusion protein-based vaccine, M72/AS01E, is a candidate that showed high efficacy in a phase 2b trial concluded in 2018 by preventing tuberculosis in adult patients which were negative for HIV with latent tuberculosis infection in the absence of active disease (NCT01755598) [55]. A more recent report confirmed that M72/AS01E protected against tuberculosis disease in about 50% of patients following 36 months of treatment. As a proof of concept, a single dose of M72/AS01E induced an increase in specific antibodies and CD4+ T cells, which was prolonged over the entire trial [56]. ID93 + GLA-SE is a recombinant subunit vaccine that has been recently tested in a phase 2 trial involving individuals vaccinated with BCG when both infected and non-infected with *M. tuberculosis*. (NCT02465216) [57]. In a preclinical study carried out on mice, this candidate vaccine induced a CD4 T cell response and a decreased number of bacteria in the lungs of animals infected with resistant or virulent strains of *M. tuberculosis*, confirming their potential as a promising candidate for human vaccine [58]. In the randomized double-blind study above mentioned, a strong antibody response was shown after two injections of ID93 + GLA-SE, which lasted throughout 6 months of trials. This response was not further boosted following a third administration. Additionally, it was well tolerated, and no serious adverse effects were noticed [57]. Further evaluation of this candidate is needed to contrast the transmission of tuberculosis disease.

### 5.2. Group B

#### 5.2.1. Extraintestinal Pathogenic *Escherichia coli* (ExPEC)

**Figure 3:**
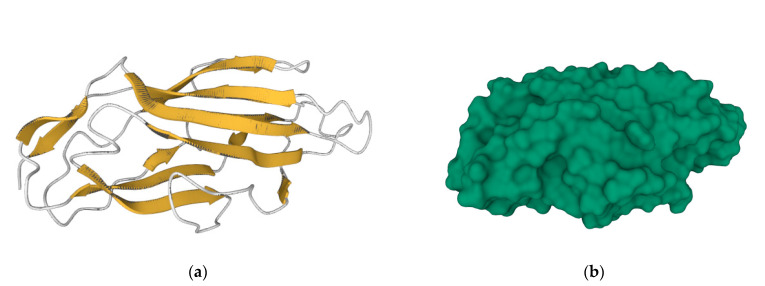
Secondary structures (**a**) and molecular surface (**b**) representation of FimH adhesin carbohydrate-binding domain from *E. coli* (https://www.rcsb.org/structure/3ZPD, accessed on 14 December 2022). Bacterial attachment involves the molecular recognition between the FimH protein surface and the mammalian cell receptor, and thus the prophylactic vaccination with FimH adhesin can impede bacterial colonization, block infection, and consequently, prevent disease.

Four candidate vaccines are under preclinical investigations against ExPEC. Two of these also target ETEC, while another one is also directed against *K. pneumoniae*. ExPEC9V is among the four candidates actually enrolled in clinical trials to counteract the invasive Extraintestinal Pathogenic *Escherichia Coli* Disease (IED) caused by ExPEC. IED affects mainly adults and old people (greater than 60 years old) who can develop sepsis and bacteremia. ExPEC9V is a nine-valent O-polysaccharide conjugate vaccine that is currently under examination in a phase 3 study, starting in 2021 and scheduled to finish within 2027 (NCT04899336, https://clinicaltrials.gov/ct2/show/NCT04899336, accessed on 9 December 2022). ExPEC10V is a polysaccharide conjugate vaccine consisting of 10 serotypes of the ExPEC that are separately conjugated to the carrier protein. This candidate has been evaluated in a phase 1 study involving Japanese patients between 60 and 85 years old with the purpose of assessing the safety and reactivity of different doses of ExPEC10V (NCT04306302 https://clinicaltrials.gov/ct2/show/NCT04306302, accessed on 9 December 2022). It is currently involved in a phase 1/2 clinical trial, which aims to evaluate the tolerability and efficacy of three different doses of ExPEC10V to further choose the optimal dose for future investigations (NCT03819049, https://clinicaltrials.gov/ct2/show/NCT03819049, accessed on 9 December 2022). Another candidate vaccine is based on FimH (Figure 3), and an adhesin protein present in the type 1 pili of *E. coli*, which is crucial in promoting the attachment on mammalian bladders.

The FimH vaccine has recently concluded a phase 1 trial with the purpose of describing the tolerability and immunogenicity of different dosages of the vaccine in 67 women, either with or without stories of urinary tract infection [59]. As a result of the good outcomes obtained so far, the FimH-based vaccine will be enrolled in a phase 2 study. A recent prophylactic strategy against ExPEC is represented by Uro-Vaxom^®^ (OM Pharma, Geneva, Switzerland), made by membrane proteins of heat-inactivated *E. coli* that are able to stimulate, not specifically, the cells of the innate immune system. This vaccine is actually evaluated in a phase 2 trial for the assessment of the prevention of urinary infection in participants with chronic neurogenic bladder dysfunction (NCT02591901) [60].

#### 5.2.2. *Salmonella enterica ser. Paratyphi A*

*S. enterica ser. Typhi,* rather than *ser. Paratyphi A* is generally assumed to cause more severe enteric fever. Remarkably, no great efforts were made in developing a vaccine against *ser. A* due to low commercial interests. However, the number of cases following *ser*. A has been widely increased over the last 20 years, especially in the population of Asia. Noteworthy, more than half (64%) of enteric fever cases are due to *ser. A* infections have been found in southeast China. Additionally, European travelers directed to Eastern countries where the infections of *ser. A* is endemic hold a high risk of spreading the disease to European countries, thus worsening the burden of the disease [61]. Therefore, the need for efficient vaccines against *S. enterica ser. A* is a priority. There are a few vaccine candidates enrolled in preclinical studies, and three vaccines are actually being involved in clinical trials. All of them are bivalent and target both *ser. Typhi* and *ser. A*. The candidate O:2 is a conjugate vaccine that showed immunogenicity and safety in phase 1 and 2 studies after a single dose. Although it was not able to arouse a booster response following a further dose, O:2 is currently involved in phase 3 trials [62].

CVD 1902, a live attenuated whole-cell vaccine, is a novel candidate proposed against *ser. A*. This vaccine was able to elicit a strong cell-mediated immune response, as confirmed in a phase 1 clinical study by high levels of specific IgG and/or IgA B-memory cells and the production of TNFa and IFNg found in volunteers after receiving one dose of CVD 1902 [63]. This vaccine has been licensed by the Indian company “Bharat Biotech International” and has been proposed to be commercialized together with the live oral S-typhi vaccine CVD 909, giving rise to a bivalent live oral vaccine against the two strains of *S. Typhi*. CVD 1902 is actually enrolled in a phase 2 study involving 76 individuals in good health who will receive two doses of this candidate to evaluate the resulting immune response and tolerability. This trial is scheduled to finish within 2023 (https://www.news-medical.net/news/20220426/Phase-III-trials-of-new-paratyphoid-vaccine-begin-in-Oxford.aspx accessed on 9 December 2022). The relatively poor incidence and mortality associated with *S. Paratyphi A* as well as the lack of specific animal models to study this infectious disease represents a big challenge in the development of an efficacy vaccine against paratyphoid.

#### 5.2.3. *Neisseria gonorrhoeae*

*N. gonorrhoeae* is a major human concern and is responsible for more than 100 million cases per year. Great efforts have been made by companies and researchers in recent decades to identify and produce a safe vaccine to prevent and treat *N. gonorrhoeae*. However, there are no licensed vaccines against this pathogen due to many difficulties. In particular, the antigenic diversity involving the surface proteins of *N. gonorrhoeae*, a knowledge gap regarding the type of necessary immune response, and the limited number of animal models mimicking the infectious disease are the main limiting factors affecting the vaccine production against this pathogen [64]. A few candidates have been identified and investigated in preclinical phases, showing promising results, including a reduced duration of infections in mice, a Type 1 T helper (Th1) response, and the production of bactericidal antibodies [65]. The retrospective case–control trial by Petousis-Harris et al. suggested the possibility that the vaccination developed for serogroup *B Neisseria meningitidis* might induce a cross-protection against *N. gonorrhoeae.* The proof of concept came from a mass vaccination against meningococcal group B executed in New Zealand with an outer membrane vesicle (OMV)-type vaccine (MeNZB). Interestingly, individuals receiving the MeNZB vaccine showed a lower chance of developing gonorrhoeae compared to not vaccinated participants (41% vs. 51%). The efficacy of this vaccine was estimated to be 31% following adjustments of gender, ethnicity, and geographical area [66]. A further study performed in New Zealand two years later demonstrated moderate effectiveness (24%) of a MeNZB vaccine in reducing hospitalization, specifically against *gonorrhoeae* [67]. These data are not surprising because *N. meningitidis* and *N. gonorrhoeae* hold at least an 80% nucleotide sequence identity. Moreover, many antigens of meningococcal B OMVs are conserved in *N. gonorrhoeae* [68]. Overall, this represents the first evidence of the effectiveness of vaccines in exerting a protective effect against *N. gonorrhoeae*.

#### 5.2.4. *Clostridioides Difficile*

**Figure 4:**
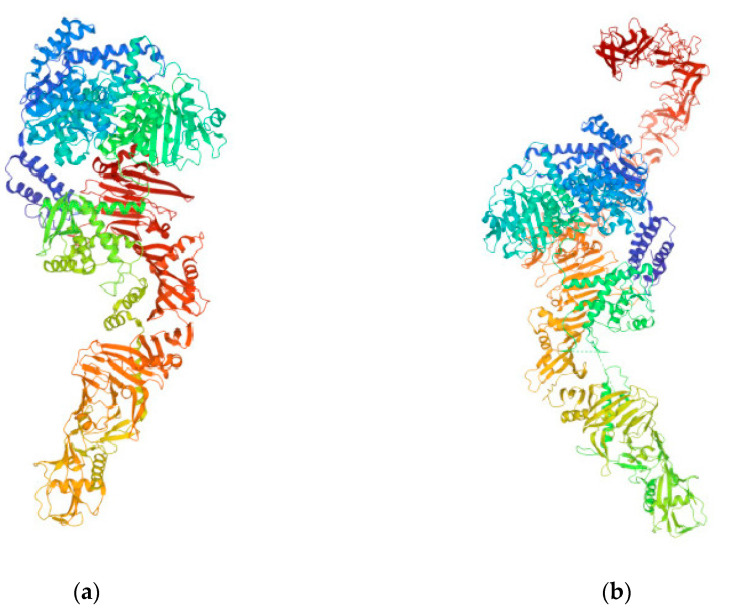
TcdA (**a**) and TcdB (**b**) from *C. difficile* (https://www.rcsb.org/3d-view/4R04 and https://www.rcsb.org/structure/6OQ5, respectively; both links were accessed on 14 December 2022). These toxins disrupt the tight junctions and the cytoskeletal structure of target human cells, which ultimately leads to cell death, and serve as targets for *C. difficile* vaccines.

*C. difficile*, formerly known as *Clostridium difficile*, is a Gram-positive bacterium responsible for *C. difficile* infection (CDI), an infectious disease characterized by symptoms such as diarrhea, colitis, and abdominal pain. CDI occurs following the ingestion of vegetative spores released by this pathogen and their spread in the gastrointestinal tract. CDI, which mainly spreads in hospitals and nursing homes, represents a major health concern as the incidence of the disease and the resulting hospitalization have raised significantly over the years [69]. *A* vaccine against *C. difficile* is not currently available on the market. There are only five candidates in preclinical studies, and even fewer reached the clinical trials. GSK has recently evaluated in a phase 1 trial a recombinant vaccine against *C. difficile*, based on the F2 antigen with or without the adjuvant AS01B (NCT04026009). The purpose of this study is to administer the vaccine GSK2904545A to healthy adults from 18 to 45 years and from 50 to 70 years to assess the safety and reactivity profile of this vaccine candidate (https://clinicaltrials.gov/ct2/show/NCT04026009, accessed on 9 December 2022). Another recent candidate has been identified by Pfizer, which had encouraging results in a phase 3 trial (Clover) testing a vaccine (PF-06425090) on 50 years old or older adult patients at risk of developing CDI. The aim was to investigate if the vaccine was able to prevent the burden of disease in a safe and efficacy way (NCT03090191, https://clinicaltrials.gov/ct2/show/NCT03090191?term=vaccine&cond=clostridium+difficile&rank=7, accessed on 9 December 2022). Mechanistically, this vaccine targets toxin A and toxin B (TcdA and TcdB, Figure 4): the main pathogenic factors of *C. difficile*.

The research of a good candidate vaccine against *C. difficile* has been characterized by a discontinuity phase over the last 10 years. VLA84 is a recombinant vaccine containing a fusion protein of truncated forms of TcdA and TcdB that successfully completed a phase 2 study a few years ago (NCT02316470). Despite the fact that it showed immunogenicity and elicited the production of antibodies against both toxins, it was not able to cover all the variants of TcdB and target the host receptor-binding region. However, there were no further updates since 2018 (this can be verified at the link https://clinicaltrials.gov/ct2/show/NCT02316470?term=vaccine, accessed on 9 December 2022).

Another candidate, ACAM-CDIFF toxoid vaccine, was discontinued in 2018 during a phase 3 study despite its good tolerability and reactivity properties (NCT01887912). The results obtained from the trial highlighted that this vaccine was not able to prevent a primary *C. difficile* infection. Indeed, the antibody response obtained by vaccination decreased very rapidly and was not strong enough to prevent the burden of the disease. Therefore, the trial was stopped since the criteria for futility were met, and this emphasizes the challenges related to vaccine development for this pathogen [70].

### 5.3. Group C

#### 5.3.1. *Klebsiella pneumoniae*

*K. pneumoniae* is a Gram-negative opportunistic pathogen able to colonize human mucous membranes, causing sepsis, pneumoniae, meningitis, and other pathological manifestations. The spread of hyper-virulent multi-drug resistant strains (MDR), such as the extended-spectrum beta-lactamase (ESBL)-producing one and *K. pneumoniae carbapenemase* (KPC), raised a global concern as these strains hold an augmented resistance to antimicrobials and they can diffuse very easily compared to other Enterobacterales. This scenario is made worse by the emergence of *K. pneumoniae* infections involving newborns in developing countries, associated with high mortality risk [71,72]. At present, no vaccines are available against *K. pneumoniae*. Five candidates have been identified in preclinical studies, while only one vaccine reached clinical trials. The tetravalent bioconjugate vaccine (Kleb4V) has recently been involved in a phase 1 trial on healthy humans to assess its safety and immunogenicity profile. Kleb4V, which includes O-antigen-polysaccharides of the most prevalent *K. pneumoniae* serotypes, is administered in four formulations, including a target dose or a low dose with or without the adjuvant AS03. The efficacy of the vaccine is evaluated in young adults (18 to 40 years old), followed by the oldest part of the population (55 to 70 years old, https://clinicaltrials.gov/ct2/show/NCT04959344, accessed on 9 December 2022).

Uromone^®^ is an inactivated enterobacterial strains-based vaccine that is commercially available and produced in Spain [73]. Uromone^®^ showed protection against recurrent urinary tract infections (UTIs) in female participants of phase 2/3 studies (NCT04096820; NCT02543827).

#### 5.3.2. *Non-typhoidal Salmonella*

NTS is a global threat and a main cause of bacteremia and meningitis in infants and adults of low-income countries and occasionally in people of industrialized countries. No safe and efficient vaccine is currently available on the market. The poor number of candidate vaccines enrolled in preclinical studies against NTS highlights the limited resources available to develop an effective vaccine despite the high mortality rate associated with this pathogen. Previously, good results have been shown following the usage of live attenuated and subunit vaccines targeting O-polysaccharides (OPS), flagellin, and other surface proteins of NTS strains, including *Salmonella enterica* subsp. *enterica ser. Typhimurium* and *Salmonella enterica* subsp*. enterica ser. Enteritidis* [74]. CVD1000 is a trivalent candidate vaccine targeting non-typhoidal *S. enterica ser. Typhimurium*, *Enteritidis,* and *Typhi*. This vaccine is enrolled in a phase 1 study with the purpose of evaluating the corresponding immune response and tolerability following the administration of different doses of CVD1000 (NCT03981952, https://clinicaltrials.gov/ct2/show/NCT03981952, accessed on 9 December 2022). Over the last year, new vaccines based on generalized modules for membrane antigens (GMMA) have been proposed to fight the burden of NTF. GMMA are bacteria-derived vesicles that are able to induce a robust immune response following the presentation of key antigens to the immune system. GMMA can be genetically modified to control undesirable immunoreactivity and to play a role as the delivery system for protein antigens [75]. GMMA targeting many pathogens have been proven to elicit a good immune response and protection without particular adverse effects both in animal models and in clinical trials [76]. GMMA-based vaccines against *S. sonnei* [77] and meningococcus [78] represent the most advanced vaccines in development in preclinical and clinical studies. The promising results obtained demonstrate the utility and efficacy of this novel strategy. The GMMA approach has been used to generate a vaccine against NTS. The work of Schager et al. showed that GMMA from *S. typhimurium* was able to evocate a strong immune response dependent on B cells and to produce antibodies against lipopolysaccharide (LPS) and porins [79].

#### 5.3.3. *Shigella* spp.

*Shigella* spp. is a Gram-negative pathogen causing acute gastrointestinal infections, local inflammations, and toxin production. It is responsible for more than 1 million deaths worldwide, especially in developing countries, with 60% involving infants [80]. Despite no vaccines still being available for *Shigella*, this is a very intense area of research, as confirmed by the presence of multiple candidates both in preclinical and in phase 1/2 clinical trials. The vaccines against *Shigella* that have been enrolled for clinical studies target both *S. flexneri* and *sonnei* and include whole cell, bioconjugate, live attenuated, subunit, and GMMA-based vaccines. A very recent candidate that is being tested is CVD31000 (CVD 1208S-122): a bivalent live attenuated vaccine targeting ETEC and *S. flexneri*. The current phase 1 trial started in September 2021 and has the purpose to assess whether the oral vaccine is safe and immunogenic in three vaccine dosage cohorts involving 54 participants between 18 and 49 years. (NCT04634513). This study is scheduled to finish within September 2024 (https://clinicaltrials.gov/ct2/show/NCT04634513 accessed on 9 December 2022). The novel monovalent bioconjugate Flexyn2a vaccine, composed of the polysaccharide component of the *S. flexneri* 2a O-antigen, was previously evaluated in a phase 1 study in 2017, demonstrating a good safety profile associated with a robust immune response (NCT02388009) [81]. A recent phase 2 study published by Talaat et al. confirmed that the Flexyn2a vaccine was well tolerated, immunogenic, and protected against *Shigella* in healthy adults. In particular, vaccinated participants exhibited protection efficacy in about 50% of cases, thus limiting antibiotic interventions. Moreover, subjects developing shigellosis showed a lower disease severity when receiving vaccination compared to the placebo group. This study will significantly contribute to the future license of the Flexyn2a vaccine as an efficacy prophylactic agent against *Shigella* (NCT02646371) [82].

### 5.4. Group D

#### 5.4.1. *Pseudomonas aeruginosa*

The diseases related to *P. aeruginosa* are among the most severe infections worldwide, responsible for hundreds of thousands of cases in the USA, Japan, and Europe [83]. Despite efforts, there are no licensed vaccines nor candidates in active clinical trials against this pathogen. The vaccines that reached clinical studies in recent years were discontinued or failed to avoid high mortality risks, as demonstrated by the IC43 recombinant *P. aeruginosa* vaccine (NCT01563263) [84]. A serious challenge for the development of an efficient vaccine against *P. aeruginosa* is represented by the high heterogeneity of proteins among the different strains of this bacterium [85].

Most vaccines produced so far to treat *P. aeruginosa* make use of antigens targeting single virulence mechanisms, including the OprF and OprI proteins [86]. Given the multiple virulence mechanisms used by *P. aeruginosa* and its ability to adapt to several host environments, other strategies exploiting the combination of multiple candidates should be considered. As shown in the work of Bianconi et al., the combination of specific candidates, rather than a single antigen, was able to control the burst of *P. aeruginosa* infections in a mouse model of respiratory infection. In particular, the synergic combination of PA5340 and PA3526-MotY proteins gave the maximum result in terms of protection, which could be used to develop an efficient vaccine against this pathogen [87]. More research to better elucidate the molecular basis of the pathogenesis of *P. aeruginosa* infections, as well as the development of in vivo models that mimick human diseases more closely, is critical to developing new potential vaccines.

#### 5.4.2. *Staphylococcus aureus*

*S. aureus* is one of the most frequent pathogens responsible for nosocomial infections. No vaccine is available against *S. aureus*. However, 14 candidates are involved in preclinical studies, and at least 2 vaccines are being tested in clinical trials. StaphVAX is a conjugate vaccine targeting capsular polysaccharide type 5 (CP5) and 8 (CP8) of *S. aureus* and was tested to prevent bacteremia in patients affected by the end-stage renal disease (ESRD). Although it showed a strong antibody response after the first vaccination, this candidate failed to show long-term efficacy, probably due to the impairment of the immune system relating to hemodialysis. Indeed, patients with ESRD typically experience reduced antigen-presenting cells and T-lymphocytes [88]. V710, a vaccine containing the *S. aureus* iron surface determinant B, which showed protection in rhesus macaques and murine models of sepsis [89], was tested in patients undergoing cardiothoracic surgery to evaluate the prevention of postoperative *S. aureus* infection. The outcome of this study revealed that the V710 vaccine did not prevent postoperative *S. aureus* infections compared to the placebo [90].

The reasons for the failure of potential vaccine development against *S. aureus* include the challenge to find an antigen target ensuring protection, the lack of efficacy in humans after positive results on animal models, and the wide range of syndromes caused by this pathogen. The development of new vaccines is challenging in virtue of scientific and economic obstacles. Suitable target validation and the duration of immunity represent the most common issues encountered during the preclinical development of vaccines. For instance, it is difficult to find appropriate targets in *S. aureus* with a resulting low-term immune response. Additionally, the mechanisms of immunity of some pathogens, such as *E. faecium*, are not elucidated yet, adding complexity and variability to vaccine development. A further complication occurs during the commercialization phase. Indeed, the approval process is generally long because of the high costs of experimentation and the uncertainness of the long-term effectiveness of vaccines. Consequently, private companies are not encouraged to invest in vaccine production because of the high financial risk. Therefore, most investments come from the public sector and philanthropic donations.

## 6. Next Generation Vaccines

The implementation of new methodologies as well as the optimization of existing procedures has always been an imperative necessity for biomedical researchers in order to ameliorate the results previously obtained and expand their current knowledge in the therapy and prophylaxis of different diseases. Therefore, old protocols for in vivo and in vitro experiments have been progressively optimized or substituted by new ones. In other cases, a synergic combination of more approaches has been useful to elucidate many biological processes [91,92,93,94,95]. The great advancement in the fields of genomics, bioinformatics, structural biology, and immunology offered new tools to improve the efficacy of new vaccine formulations against the AMR. These promising strategies include bioconjugation, reverse vaccinology, and novel adjuvants. Bioconjugation represents a novel technology in the field of immunology that permits the generation of conjugate vaccines in a biological environment, often represented by bacteria, thus preserving the native immunogenic structures. Bioconjugates are molecules composed of carrier proteins covalently linked to polysaccharide chains to elicit a strong immune response. This mechanism frequently occurs in vivo in *E. coli*, where the specific enzyme PglB permits the link between the glycan and specific moieties of the proteins. This strategy, which is advantageous in terms of rapidity, reproducibility, and costs, has been mostly used to generate potential vaccines directly against Gram-negative pathogens, including *Shigella* [96] and *K. pneumoniae* [97]. The concept behind reverse vaccinology is to find exposed surface proteins with a pathogenic role that can stimulate the immune system. The identification of such proteins occurs by whole genome sequencing approaches rather than searching from microbe cultures [98]. Once the pathogen sequence is identified, the revealed proteins can be analyzed by using bioinformatic tools focusing on those ones predicted to be exposed on the surface. These proteins are thus transformed in bacteria, such as *E. coli*, which are purified and tested as potential candidate vaccines [99]. This strategy has been used to develop successfully meningococcus B [98], *E. coli* [100], and, more recently, *P. aeruginosa* vaccines [87]. The adjuvants are known to be crucial for sharpening the immune response induced by vaccines. Nonetheless, many adjuvants contained in vaccines have issues related to stability, tolerability, safety, or lack of efficacy. The development of novel adjuvants relies on new formulations based on micro- and nanoparticulate platforms, such as liposomes, aluminum salts, and emulsions. AS01 is a liposome formulation that showed strong efficacy with an improved T helper cell-mediated response and humoral immunity in mice as well as humans. Indeed, this adjuvant has recently been licensed for a vaccine against malaria [101] as well as to treat Herpes Zoster [102]. Saponin QS21 and monophosphoryl lipid (MPL) formulated in liposomes, such as AS01, act synergically as immunostimulatory molecules to elicit a strong immune response.

## 7. Conclusions

AMR is a global emergency that is increasing the mortality risk and negatively affecting related medical costs, especially in low and medium-income countries. The development of novel antibiotics alone is not an effective strategy to overcome the AMR burden because of the enormous challenges encountered by researchers and companies involved in this field. Vaccines directed against bacterial diseases may play a crucial role in limiting the threat of AMR. Indeed, they can directly eradicate the pathogen, lowering the incidence of infections and avoiding, in turn, the necessity of antibiotics. Moreover, vaccines directed against viruses also inhibit secondary bacterial infections, reducing the inappropriate consumption of antibiotics. As explained above, avoiding the misuse of antibiotics in the veterinary field is also crucial to avoid the high mortality rates observed in animals and the transmission of resistant pathogens to humans. Despite the huge efforts made so far by the scientific community, vaccines for most of the important pathogens are not available yet. This is due to the main issues typically found during vaccine development, including the lack of specific animal models mimicking the disease, the complexity of some bacterial pathogens, and the difficulties related to the efficacy and safety of novel vaccines. However, a significant number of candidates are being tested both in preclinical and clinical trials. Moreover, there is an advanced comprehension of the molecular mechanisms by which pathogens act and a more detailed vision of the immune system compared to the past. The new-generation vaccines, which exploit novel strategies such as reverse vaccinology, bioconjugates, and new adjuvants, may represent a key factor for the successful production of potential vaccines. In conclusion, a synergic approach involving the research of new antibiotics and the improvement of vaccine production is required to counteract efficiently AMR occurrence.

## Figures and Tables

**Table 1:** Effects of vaccination on the transmission rate of infections in different age groups.

Pathogen	Transmission Reduction Rate (%)	Reference	Age (Years)
*Streptococcus pneumonia*	84	[30]	<2
*Streptococcus pneumonia*	49	[30]	65
Influenza virus A and B	47	[32]	0.5–2.9

**Table 2:** Number of vaccine candidates by pathogen in preclinical and active clinical development.

Pathogens	Number of Candidates	
	Preclinical Phase	Active Clinical Phase
*Mycobacterium tuberculosis*	20	13
*Streptococcus pneumonia*	17	16
*Staphylococcus aureus*	14	2
*Shigella flexneri*	0	6
*Shigella* spp.	10	
*Enterotoxigenic Escherichia coli* (ETEC)	10	6
*Salmonella enterica ser. Typhi*	8	5
*Non-typhoidal Salmonella* (NTS)	6	1
*Helicobacter pylori*	6	0
*Klebsiella pneumoniae*	5	1
*Clostridioides difficile*	5	2
*Salmonella enterica ser Paratyphi A*	3	4
*Haemophilus influenzae* type b *(Hib)*	3	4
*Extraintestinal pathogenic Escherichia coli* (ExPEC)	4	4
*Shigella sonnei*	0	3
*Neisseria gonorrhoeae*	2	1
*Acinetobacter baumannii*	5	0
*Pseudomonas aeruginosa*	4	0
*Campylobacter jejuni*	4	0
*Enterococcus faecium*	0	0
*Enterobacter* spp.	0	0

## Data Availability

Not applicable.

## References

[1] Zinner S.H. (2007). Antibiotic use: Present and future. Microbiol. Bologna.

[2] Mohr K.I. (2016). History of Antibiotics Research. Curr. Top. Microbiol. Immunol..

[3] Jansen K.U., Knirsch C., Anderson A.S. (2018). The role of vaccines in preventing bacterial antimicrobial resistance. Nat. Med..

[4] McEwen S.A., Collignon P.J., Aarestrup F.M., Schwarz S., Shen J., Cavaco L. (2018). Antimicrobial Resistance: A One Health Perspective. Microbiol. Spectr..

[5] Okeke I.N., Aboderin O.A., Byarugaba D.K., Ojo K.K., Opintan J.A. (2007). Growing Problem of Multidrug-Resistant Enteric Pathogens in Africa. Emerg. Infect. Dis..

[6] Lansang M.A., Lucas-Aquino R., Tupasi T.E., Mina V.S., Salazar L.S., Juban N., Limjoco T.T., Nisperos L.E., Kunin C.M. (1990). Purchase of antibiotics without prescription in Manila, The Philippines. Inappropriate choices and doses. J. Clin. Epidemiol..

[7] Risha P.G., Shewiyo D., Msami A., Masuki G., Vergote G., Vervaet C., Remon J.P. (2002). In vitro evaluation of the quality of essential drugs on the Tanzanian market. Trop. Med. Int. Health.

[8] Basco L.K. (2004). Molecular epidemiology of malaria in Cameroon. XIX. Quality of antimalarial drugs used for self-medication. Am. J. Trop. Med. Hyg..

[9] Saleh N., Awada S., Awwad R., Jibai S., Arfoul C., Zaiter L., Dib W., Salameh P. (2015). Evaluation of antibiotic prescription in the Lebanese community: A pilot study. Infect. Ecol. Epidemiol..

[10] Kumar A., Roberts D., Wood K.E., Light B., Parrillo J.E., Sharma S., Suppes R., Feinstein D., Zanotti S., Taiberg L. (2006). Duration of hypotension before initiation of effective antimicrobial therapy is the critical determinant of survival in human septic shock *. Crit. Care Med..

[11] Khan Z.A., Siddiqui M.F., Park S. (2019). Current and Emerging Methods of Antibiotic Susceptibility Testing. Diagnostics.

[12] March Rosselló G.A., García-Loygorri Jordán de Urries M.C., Gutiérrez Rodríguez M.P., Simarro Grande M., Orduña Domingo A., Bratos Pérez M.Á. (2016). A two-hour antibiotic susceptibility test by ATP-bioluminescence. Enferm. Infecc. Y Microbiol. Clín..

[13] Neu H.C. (1992). The Crisis in Antibiotic Resistance. Science.

[14] Ayukekbong J.A., Ntemgwa M., Atabe A.N. (2017). The threat of antimicrobial resistance in developing countries: Causes and control strategies. Antimicrob. Resist. Infect. Control.

[15] Witte W. (1998). Medical Consequences of Antibiotic Use in Agriculture. Science.

[16] Angulo F.J., Collignon P., Wegener H.C., Braam P., Butler C.D. (2005). The Routine Use of Antibiotics to Promote Animal Growth Does Little to Benefit Protein Undernutrition in the Developing World. Clin. Infect. Dis..

[17] Mitema E.S., Kikuvi G.M., Wegener H.C., Stohr K. (2002). An assessment of antimicrobial consumption in food producing animals in Kenya. J. Vet. Pharmacol. Ther..

[18] Mezali L., Hamdi T.M. (2012). Prevalence and Antimicrobial Resistance of Salmonella Isolated from Meat and Meat Products in Algiers (Algeria). Foodborne Pathog. Dis..

[19] Kapoor G., Saigal S., Elongavan A. (2017). Action and resistance mechanisms of antibiotics: A guide for clinicians. J. Anaesthesiol. Clin. Pharmacol..

[20] Brooks B.D., Brooks A.E. (2014). Therapeutic strategies to combat antibiotic resistance. Adv. Drug Deliv. Rev..

[21] Baker S.J., Payne D.J., Rappuoli R., De Gregorio E. (2018). Technologies to address antimicrobial resistance. Proc. Natl. Acad. Sci. USA.

[22] Payne D.J., Miller L.F., Findlay D., Anderson J., Marks L. (2015). Time for a change: Addressing R&D and commercialization challenges for antibacterials. Philos. Trans. R. Soc. B Biol. Sci..

[23] Payne D.J., Gwynn M.N., Holmes D.J., Pompliano D.L. (2006). Drugs for bad bugs: Confronting the challenges of antibacterial discovery. Nat. Rev. Drug Discov..

[24] Lode H. (2010). Safety and tolerability of commonly prescribed oral antibiotics for the treatment of respiratory tract infections. Am. J. Med..

[25] Renwick M., Mossialos E. (2018). What are the economic barriers of antibiotic R&D and how can we overcome them?. Expert Opin. Drug Discov..

[26] Micoli F., Bagnoli F., Rappuoli R., Serruto D. (2021). The role of vaccines in combatting antimicrobial resistance. Nat. Rev. Microbiol..

[27] Costanzo M., De Giglio M.A.R., Roviello G.N. (2022). Anti-Coronavirus Vaccines: Past Investigations on SARS-CoV-1 and MERS-CoV, the Approved Vaccines from BioNTech/Pfizer, Moderna, Oxford/AstraZeneca and others under Development Against SARSCoV-2 Infection. Curr. Med. Chem..

[28] Farhud D.D., Zokaei S. (2021). A Brief Overview of COVID-19 Vaccines. Iran. J. Public Health.

[29] Bagnoli F., Payne D.J. (2017). Reaction: Alternative Modalities to Address Antibiotic-Resistant Pathogens. Chem.

[30] Committee N.V.A. (2016). A call for greater consideration for the role of vaccines in national strategies to combat antibiotic-resistant bacteria: Recommendations from the national vaccine advisory committee: Approved by the National Vaccine Advisory Committee on 10 June 2015. Public Health Rep..

[31] Kennedy D.A., Read A.F. (2018). Why the evolution of vaccine resistance is less of a concern than the evolution of drug resistance. Proc. Natl. Acad. Sci. USA.

[32] Danier J., Rivera L., Claeys C., Dbaibo G., Jain V.K., Kosalaraksa P., Woo W., Yanni E., Zaman K., Acosta B. (2019). Clinical Presentation of Influenza in Children 6 to 35 Months of Age. Pediatr. Infect. Dis. J..

[33] Strassburg M.A. (1982). The global eradication of smallpox. Am. J. Infect. Control.

[34] Roeder P., Mariner J., Kock R. (2013). Rinderpest: The veterinary perspective on eradication. Philos. Trans. R. Soc. B Biol. Sci..

[35] Bandyopadhyay A.S., Garon J., Seib K., Orenstein W.A. (2015). Polio vaccination: Past, present and future. Future Microbiol..

[36] Rappuoli R., Pizza M., Del Giudice G., De Gregorio E. (2014). Vaccines, new opportunities for a new society. Proc. Natl. Acad. Sci. USA.

[37] Kwong J.C., Maaten S., Upshur R.E.G., Patrick D.M., Marra F. (2009). The Effect of Universal Influenza Immunization on Antibiotic Prescriptions: An Ecological Study. Clin. Infect. Dis..

[38] Ozgur S.K., Beyazova U., Kemaloglu Y.K., Maral I., Sahin F., Camurdan A.D., Kizil Y., Dinc E., Tuzun H. (2006). Effectiveness of Inactivated Influenza Vaccine for Prevention of Otitis Media in Children. Pediatr. Infect. Dis. J..

[39] MacLennan C.A., Martin L.B., Micoli F. (2014). Vaccines against invasive *Salmonella* disease. Hum. Vaccines Immunother..

[40] Borrow R., Medise B.E., Soedjatmiko S., Rengganis I., Gunardi H., Sekartini R., Koesno S., Satari H.I., Hadinegoro S.R., Yang J.S. (2019). Six-month follow up of a randomized clinical trial-phase I study in Indonesian adults and children: Safety and immunogenicity of Salmonella typhi polysaccharide-diphtheria toxoid (Vi-DT) conjugate vaccine. PLoS ONE.

[41] Capeding M.R., Sil A., Tadesse B.T., Saluja T., Teshome S., Alberto E., Kim D.R., Park E.L., Park J.Y., Yang J.S. (2020). Safety and immunogenicity of Vi-DT conjugate vaccine among 6–23-month-old children: Phase II, randomized, dose-scheduling, observer-blind Study. EClinicalMedicine.

[42] Baliban S., Allen J., Curtis B., Amin M., Lees A., Rao R., Naidu G., Venkatesan R., Rao D., Mohan V. (2018). Immunogenicity and Induction of Functional Antibodies in Rabbits Immunized with a Trivalent Typhoid-Invasive Nontyphoidal Salmonella Glycoconjugate Formulation. Molecules.

[43] Johnson C., Darton T.C., Jones C., Blohmke C.J., Waddington C.S., Zhou L., Peters A., Haworth K., Sie R., Green C.A. (2016). Using a Human Challenge Model of Infection to Measure Vaccine Efficacy: A Randomised, Controlled Trial Comparing the Typhoid Vaccines M01ZH09 with Placebo and Ty21a. PLoS Negl. Trop. Dis..

[44] Ryan E.T., Cartee R.T., Thanawastien A., Griffin Iv T.J., Mekalanos J.J., Bart S., Killeen K.P. (2020). A phase 1 randomized safety, reactogenicity, and immunogenicity study of Typhax: A novel protein capsular matrix vaccine candidate for the prevention of typhoid fever. PLoS Negl. Trop. Dis..

[45] Honda-Okubo Y., Cartee R.T., Thanawastien A., Seung Yang J., Killeen K.P., Petrovsky N. (2022). A typhoid fever protein capsular matrix vaccine candidate formulated with Advax-CpG adjuvant induces a robust and durable anti-typhoid Vi polysaccharide antibody response in mice, rabbits and nonhuman primates. Vaccine.

[46] Trunz B.B., Fine P.E.M., Dye C. (2006). Effect of BCG vaccination on childhood tuberculous meningitis and miliary tuberculosis worldwide: A meta-analysis and assessment of cost-effectiveness. Lancet.

[47] Gamez-Gonzalez L.B., Hamada H., Llamas-Guillen B.A., Ruiz-Fernandez M., Yamazaki-Nakashimada M. (2017). BCG and Kawasaki disease in Mexico and Japan. Hum. Vaccines Immunother..

[48] Davids V., Hanekom W.A., Mansoor N., Gamieldien H., Gelderbloem S.J., Hawkridge A., Hussey G.D., Hughes E.J., Soler J., Murray R.A. (2006). The Effect of Bacille Calmette-Guérin Vaccine Strain and Route of Administration on Induced Immune Responses in Vaccinated Infants. J. Infect. Dis..

[49] Manissero D., Lopalco P.L., Levy-Bruhl D., Ciofi degli Atti M.L., Giesecke J. (2008). Assessing the impact of different BCG vaccination strategies on severe childhood TB in low-intermediate prevalence settings. Vaccine.

[50] Spertini F., Audran R., Chakour R., Karoui O., Steiner-Monard V., Thierry A.-C., Mayor C.E., Rettby N., Jaton K., Vallotton L. (2015). Safety of human immunisation with a live-attenuated Mycobacterium tuberculosis vaccine: A randomised, double-blind, controlled phase I trial. Lancet Respir. Med..

[51] Tameris M., Mearns H., Penn-Nicholson A., Gregg Y., Bilek N., Mabwe S., Geldenhuys H., Shenje J., Luabeya A.K.K., Murillo I. (2019). Live-attenuated Mycobacterium tuberculosis vaccine MTBVAC versus BCG in adults and neonates: A randomised controlled, double-blind dose-escalation trial. Lancet Respir. Med..

[52] White A.D., Sibley L., Sarfas C., Morrison A., Gullick J., Clark S., Gleeson F., McIntyre A., Arlehamn C.L., Sette A. (2021). MTBVAC vaccination protects rhesus macaques against aerosol challenge with M. tuberculosis and induces immune signatures analogous to those observed in clinical studies. NPJ Vaccines.

[53] Martín C., Marinova D., Aguiló N., Gonzalo-Asensio J. (2021). MTBVAC, a live TB vaccine poised to initiate efficacy trials 100 years after BCG. Vaccine.

[54] Sharma S.K., Katoch K., Sarin R., Balambal R., Kumar Jain N., Patel N., Murthy K.J.R., Singla N., Saha P.K., Khanna A. (2017). Efficacy and Safety of Mycobacterium indicus pranii as an adjunct therapy in Category II pulmonary tuberculosis in a randomized trial. Sci. Rep..

[55] Van Der Meeren O., Hatherill M., Nduba V., Wilkinson R.J., Muyoyeta M., Van Brakel E., Ayles H.M., Henostroza G., Thienemann F., Scriba T.J. (2018). Phase 2b Controlled Trial of M72/AS01EVaccine to Prevent Tuberculosis. N. Engl. J. Med..

[56] Tait D.R., Hatherill M., Van Der Meeren O., Ginsberg A.M., Van Brakel E., Salaun B., Scriba T.J., Akite E.J., Ayles H.M., Bollaerts A. (2019). Final Analysis of a Trial of M72/AS01E Vaccine to Prevent Tuberculosis. N. Engl. J. Med..

[57] Day T.A., Penn-Nicholson A., Luabeya A.K.K., Fiore-Gartland A., Du Plessis N., Loxton A.G., Vergara J., Rolf T.A., Reid T.D., Toefy A. (2021). Safety and immunogenicity of the adjunct therapeutic vaccine ID93 + GLA-SE in adults who have completed treatment for tuberculosis: A randomised, double-blind, placebo-controlled, phase 2a trial. Lancet Respir. Med..

[58] Bertholet S., Ireton G.C., Ordway D.J., Windish H.P., Pine S.O., Kahn M., Phan T., Orme I.M., Vedvick T.S., Baldwin S.L. (2010). A Defined Tuberculosis Vaccine Candidate Boosts BCG and Protects Against Multidrug-Resistant *Mycobacterium tuberculosis*. Sci. Transl. Med..

[59] Eldridge G.R., Hughey H., Rosenberger L., Martin S.M., Shapiro A.M., D’Antonio E., Krejci K.G., Shore N., Peterson J., Lukes A.S. (2020). Safety and immunogenicity of an adjuvanted Escherichia coli adhesin vaccine in healthy women with and without histories of recurrent urinary tract infections: Results from a first-in-human phase 1 study. Hum. Vaccines Immunother..

[60] Wade D., Cooper J., Derry F., Taylor J. (2019). Uro-Vaxom^®^ versus placebo for the prevention of recurrent symptomatic urinary tract infections in participants with chronic neurogenic bladder dysfunction: A randomised controlled feasibility study. Trials.

[61] Kuijpers L.M.F., Le Hello S., Fawal N., Fabre L., Tourdjman M., Dufour M., Sar D., Kham C., Phe T., Vlieghe E. (2016). Genomic analysis of *Salmonella enterica* serotype Paratyphi A during an outbreak in Cambodia, 2013–2015. Microb. Genom..

[62] Martin L.B., Simon R., MacLennan C.A., Tennant S.M., Sahastrabuddhe S., Khan M.I. (2016). Status of paratyphoid fever vaccine research and development. Vaccine.

[63] Wahid R., Kotloff K.L., Levine M.M., Sztein M.B. (2019). Cell mediated immune responses elicited in volunteers following immunization with candidate live oral *Salmonella enterica* serovar Paratyphi A attenuated vaccine strain CVD 1902. Clin. Immunol..

[64] Edwards J.L., Jennings M.P., Seib K.L. (2018). Neisseria gonorrhoeae vaccine development. Curr. Opin. Infect. Dis..

[65] Rice P.A., Shafer W.M., Ram S., Jerse A.E. (2017). Neisseria gonorrhoeae: Drug Resistance, Mouse Models, and Vaccine Development. Annu. Rev. Microbiol..

[66] Petousis-Harris H., Paynter J., Morgan J., Saxton P., McArdle B., Goodyear-Smith F., Black S. (2017). Effectiveness of a group B outer membrane vesicle meningococcal vaccine against gonorrhoea in New Zealand: A retrospective case-control study. Lancet.

[67] Paynter J., Goodyear-Smith F., Morgan J., Saxton P., Black S., Petousis-Harris H. (2019). Effectiveness of a Group B Outer Membrane Vesicle Meningococcal Vaccine in Preventing Hospitalization from Gonorrhea in New Zealand: A Retrospective Cohort Study. Vaccines.

[68] Semchenko E.A., Tan A., Borrow R., Seib K.L. (2019). The Serogroup B Meningococcal Vaccine Bexsero Elicits Antibodies to Neisseria gonorrhoeae. Clin. Infect. Dis..

[69] Johnson S., Gerding D.N. (1998). Clostridium difficile–Associated Diarrhea. Clin. Infect. Dis..

[70] De Bruyn G., Gordon D.L., Steiner T., Tambyah P., Cosgrove C., Martens M., Bassily E., Chan E.-S., Patel D., Chen J. (2021). Safety, immunogenicity, and efficacy of a Clostridioides difficile toxoid vaccine candidate: A phase 3 multicentre, observer-blind, randomised, controlled trial. Lancet Infect. Dis..

[71] Ballot D.E., Bandini R., Nana T., Bosman N., Thomas T., Davies V.A., Cooper P.A., Mer M., Lipman J. (2019). A review of -multidrug-resistant Enterobacteriaceae in a neonatal unit in Johannesburg, South Africa. BMC Pediatr..

[72] Bassetti M., Righi E., Carnelutti A., Graziano E., Russo A. (2018). Multidrug-resistant *Klebsiella pneumoniae*: Challenges for treatment, prevention and infection control. Expert Rev. Anti-Infect. Ther..

[73] Lorenzo-Gómez M.F., Padilla-Fernández B., García-Criado F.J., Mirón-Canelo J.A., Gil-Vicente A., Nieto-Huertos A., Silva-Abuin J.M. (2012). Evaluation of a therapeutic vaccine for the prevention of recurrent urinary tract infections versus prophylactic treatment with antibiotics. Int. Urogynecol. J..

[74] Tennant S.M., MacLennan C.A., Simon R., Martin L.B., Khan M.I. (2016). Nontyphoidal salmonella disease: Current status of vaccine research and development. Vaccine.

[75] Mancini F., Micoli F., Necchi F., Pizza M., Berlanda Scorza F., Rossi O. (2021). GMMA-Based Vaccines: The Known and The Unknown. Front. Immunol..

[76] Micoli F., Rondini S., Alfini R., Lanzilao L., Necchi F., Negrea A., Rossi O., Brandt C., Clare S., Mastroeni P. (2018). Comparative immunogenicity and efficacy of equivalent outer membrane vesicle and glycoconjugate vaccines against nontyphoidal Salmonella. Proc. Natl. Acad. Sci. USA.

[77] Liu Y., Hammer L.A., Liu W., Hobbs M.M., Zielke R.A., Sikora A.E., Jerse A.E., Egilmez N.K., Russell M.W. (2017). Experimental vaccine induces Th1-driven immune responses and resistance to Neisseria gonorrhoeae infection in a murine model. Mucosal Immunol..

[78] Keiser P.B., Gibbs B.T., Coster T.S., Moran E.E., Stoddard M.B., Labrie J.E., Schmiel D.H., Pinto V., Chen P., Zollinger W.D. (2010). A phase 1 study of a group B meningococcal native outer membrane vesicle vaccine made from a strain with deleted lpxL2 and synX and stable expression of opcA. Vaccine.

[79] Schager A.E., Dominguez-Medina C.C., Necchi F., Micoli F., Goh Y.S., Goodall M., Flores-Langarica A., Bobat S., Cook C.N.L., Arcuri M. (2018). IgG Responses to Porins and Lipopolysaccharide within an Outer Membrane-Based Vaccine against Nontyphoidal Salmonella Develop at Discordant Rates. mBio.

[80] Ashkenazi S., Cohen D. (2013). An update on vaccines against Shigella. Ther. Adv. Vaccines.

[81] Riddle M.S., Kaminski R.W., Di Paolo C., Porter C.K., Gutierrez R.L., Clarkson K.A., Weerts H.E., Duplessis C., Castellano A., Alaimo C. (2016). Safety and Immunogenicity of a Candidate Bioconjugate Vaccine against *Shigella flexneri* 2a Administered to Healthy Adults: A Single-Blind, Randomized Phase I Study. Clin. Vaccine Immunol..

[82] Talaat K.R., Alaimo C., Martin P., Bourgeois A.L., Dreyer A.M., Kaminski R.W., Porter C.K., Chakraborty S., Clarkson K.A., Brubaker J. (2021). Human challenge study with a Shigella bioconjugate vaccine: Analyses of clinical efficacy and correlate of protection. EBioMedicine.

[83] Boucher H.W., Talbot G.H., Bradley J.S., Edwards J.E., Gilbert D., Rice L.B., Scheld M., Spellberg B., Bartlett J. (2009). Bad Bugs, No Drugs: No ESKAPE! An Update from the Infectious Diseases Society of America. Clin. Infect. Dis..

[84] Adlbrecht C., Wurm R., Depuydt P., Spapen H., Lorente J.A., Staudinger T., Creteur J., Zauner C., Meier-Hellmann A., Eller P. (2020). Efficacy, immunogenicity, and safety of IC43 recombinant Pseudomonas aeruginosa vaccine in mechanically ventilated intensive care patients—A randomized clinical trial. Crit. Care.

[85] Döring G., Pier G.B. (2008). Vaccines and immunotherapy against Pseudomonas aeruginosa. Vaccine.

[86] Westritschnig K., Hochreiter R., Wallner G., Firbas C., Schwameis M., Jilma B. (2013). A randomized, placebo-controlled phase I study assessing the safety and immunogenicity of a *Pseudomonas aeruginosa* hybrid outer membrane protein OprF/I vaccine (IC43) in healthy volunteers. Hum. Vaccines Immunother..

[87] Bianconi I., Alcalá-Franco B., Scarselli M., Dalsass M., Buccato S., Colaprico A., Marchi S., Masignani V., Bragonzi A. (2019). Genome-Based Approach Delivers Vaccine Candidates against *Pseudomonas aeruginosa*. Front. Immunol..

[88] Fattom A., Matalon A., Buerkert J., Taylor K., Damaso S., Boutriau D. (2015). Efficacy profile of a bivalent *Staphylococcus aureus* glycoconjugated vaccine in adults on hemodialysis: Phase III randomized study. Hum. Vaccines Immunother..

[89] Kuklin N.A., Clark D.J., Secore S., Cook J., Cope L.D., McNeely T., Noble L., Brown M.J., Zorman J.K., Wang X.M. (2006). A Novel Staphylococcus aureus Vaccine: Iron Surface Determinant B Induces Rapid Antibody Responses in Rhesus Macaques and Specific Increased Survival in a Murine S. aureus Sepsis Model. Infect. Immun..

[90] Fowler V.G., Allen K.B., Moreira E.D., Moustafa M., Isgro F., Boucher H.W., Corey G.R., Carmeli Y., Betts R., Hartzel J.S. (2013). Effect of an Investigational Vaccine for Preventing Staphylococcus aureus Infections after Cardiothoracic Surgery. JAMA.

[91] Costanzo V., D’Apolito L., Sardella D., Iervolino A., La Manna G., Capasso G., Frische S., Trepiccione F. (2022). Single nephron glomerular filtration rate measured by linescan multiphoton microscopy compared to conventional micropuncture. Pflügers Arch. Eur. J. Physiol..

[92] Jorrin-Novo J.V. (2020). What Is New in (Plant) Proteomics Methods and Protocols: The 2015–2019 Quinquennium. Methods Mol. Biol..

[93] Lieske A., Ha T.C., Schambach A., Maetzig T. (2021). An Improved Lentiviral Fluorescent Genetic Barcoding Approach Distinguishes Hematopoietic Stem Cell Properties in Multiplexed In Vivo Experiments. Hum. Gene Ther..

[94] McMillen P., Novak R., Levin M. (2020). Toward Decoding Bioelectric Events in Xenopus Embryogenesis: New Methodology for Tracking Interplay Between Calcium and Resting Potentials In Vivo. J. Mol. Biol..

[95] Engbjerg J.S., Costanzo V., Sardella D., Bordoni L., Jakobsen S., D’Apolito L., Frøkiær J., Trepiccione F., Capasso G., Frische S. (2022). The Probe for Renal Organic Cation Secretion (4-Dimethylaminostyryl)-N-Methylpyridinium (ASP+)) Shows Amplified Fluorescence by Binding to Albumin and Is Accumulated In Vivo. Mol. Imaging.

[96] Martin P., Alaimo C. (2022). The Ongoing Journey of a Shigella Bioconjugate Vaccine. Vaccines.

[97] Feldman M.F., Mayer Bridwell A.E., Scott N.E., Vinogradov E., McKee S.R., Chavez S.M., Twentyman J., Stallings C.L., Rosen D.A., Harding C.M. (2019). A promising bioconjugate vaccine against hypervirulent Klebsiella pneumoniae. Proc. Natl. Acad. Sci. USA.

[98] Serruto D., Bottomley M.J., Ram S., Giuliani M.M., Rappuoli R. (2012). The new multicomponent vaccine against meningococcal serogroup B, 4CMenB: Immunological, functional and structural characterization of the antigens. Vaccine.

[99] Pizza M., Scarlato V., Masignani V., Giuliani M.M., Aricò B., Comanducci M., Jennings G.T., Baldi L., Bartolini E., Capecchi B. (2000). Identification of Vaccine Candidates Against Serogroup B Meningococcus by Whole-Genome Sequencing. Science.

[100] Moriel D.G., Bertoldi I., Spagnuolo A., Marchi S., Rosini R., Nesta B., Pastorello I., Corea V.A.M., Torricelli G., Cartocci E. (2010). Identification of protective and broadly conserved vaccine antigens from the genome of extraintestinal pathogenic *Escherichia coli*. Proc. Natl. Acad. Sci. USA.

[101] Otieno L., Guerra Mendoza Y., Adjei S., Agbenyega T., Agnandji S.T., Aide P., Akoo P., Ansong D., Asante K.P., Berkley J.A. (2020). Safety and immunogenicity of the RTS,S/AS01 malaria vaccine in infants and children identified as HIV-infected during a randomized trial in sub-Saharan Africa. Vaccine.

[102] Cunningham A.L., Lal H., Kovac M., Chlibek R., Hwang S.-J., Díez-Domingo J., Godeaux O., Levin M.J., McElhaney J.E., Puig-Barberà J. (2016). Efficacy of the Herpes Zoster Subunit Vaccine in Adults 70 Years of Age or Older. N. Engl. J. Med..

